# Identification and validation of a novel cuproptosis-related stemness signature to predict prognosis and immune landscape in lung adenocarcinoma by integrating single-cell and bulk RNA-sequencing

**DOI:** 10.3389/fimmu.2023.1174762

**Published:** 2023-05-23

**Authors:** Jia Yang, Kaile Liu, Lu Yang, Junqing Ji, Jingru Qin, Haibin Deng, Zhongqi Wang

**Affiliations:** Department of Medical Oncology, Longhua Hospital Shanghai University of Traditional Chinese Medicine, Shanghai, China

**Keywords:** cuproptosis-related stemness genes (CRSGs), prognostic signature, immune landscape, single-cell sequencing, lung adenocarcinoma, cancer stem cells

## Abstract

**Background:**

Cancer stem cells (CSCs) play vital roles in lung adenocarcinoma (LUAD) recurrence, metastasis, and drug resistance. Cuproptosis has provided a novel insight into the treatment of lung CSCs. However, there is a lack of knowledge regarding the cuproptosis-related genes combined with the stemness signature and their roles in the prognosis and immune landscape of LUAD.

**Methods:**

Cuproptosis-related stemness genes (CRSGs) were identified by integrating single-cell and bulk RNA-sequencing data in LUAD patients. Subsequently, cuproptosis-related stemness subtypes were classified using consensus clustering analysis, and a prognostic signature was constructed by univariate and least absolute shrinkage operator (LASSO) Cox regression. The association between signature with immune infiltration, immunotherapy, and stemness features was also investigated. Finally, the expression of CRSGs and the functional roles of target gene were validated *in vitro*.

**Results:**

We identified six CRSGs that were mainly expressed in epithelial and myeloid cells. Three distinct cuproptosis-related stemness subtypes were identified and associated with the immune infiltration and immunotherapy response. Furthermore, a prognostic signature was constructed to predict the overall survival (OS) of LUAD patients based on eight differently expressed genes (DEGs) with cuproptosis-related stemness signature (KLF4, SCGB3A1, COL1A1, SPP1, C4BPA, TSPAN7, CAV2, and CTHRC1) and confirmed in validation cohorts. We also developed an accurate nomogram to improve clinical applicability. Patients in the high-risk group showed worse OS with lower levels of immune cell infiltration and higher stemness features. Ultimately, further cellular experiments were performed to verify the expression of CRSGs and prognostic DEGs and demonstrate that SPP1 could affect the proliferation, migration, and stemness of LUAD cells.

**Conclusion:**

This study developed a novel cuproptosis-related stemness signature that can be used to predict the prognosis and immune landscape of LUAD patients, and provided potential therapeutic targets for lung CSCs in the future.

## Introduction

The most prevalent type of lung cancer, lung adenocarcinoma (LUAD), is the primary reason for cancer-related deaths worldwide (1). Although the advances of treatment in LUAD over the past 20 years, the 5-year overall survival (OS) is still below 20% due to its high recurrence and metastasis (2, 3). Increasing evidence indicates that lung cancer stem cells (CSCs) play a critical role in LUAD, and their self-renewal, unlimited proliferation, and immunosuppressive properties are responsible for generating tumor heterogeneity and radio-chemotherapy resistance (4, 5). Despite salinomycin and its derivatives have been identified that preferentially target breast CSCs (6, 7), more efforts are needed to identify novel therapeutic targets and develop effective prognostic models for LUAD patients to break the logjam of CSCs-mediated drug resistance and immune suppression.

Since the low levels of ROS in CSCs, new therapeutic strategies for generating intracellular reactive oxygen species (ROS) by exogenous metal chelators and ionophores have emerged (8). Copper (Cu), as an essential element for accumulating ROS, is closely related to the progression of cancer by promoting proliferation, angiogenesis, metastasis, and regulating immune responses (9, 10). A series of copper complexes have demonstrated encouraging anticancer potential by selectively suppressing lung, colorectal, and breast CSCs, including copper ionophore such as disulfiram, which has already entered phase I (11, 12). Recent studies have revealed this novel copper-dependent cell death that is triggered by copper ionophores, known as cuproptosis (13), which is associated with mitochondrial respiration and the tricarboxylic acid (TCA) cycle, resulting in proteotoxic stress that is distinct from oxidative stress-related cell death (14). Since Tsvetkov et al. first proposed that FDX1, LIAS, LIPT1, DLD, DLAT, PDHA1, and PDHB are positive cuproptosis-related genes, while MTF1, GLS, and CDKN2A are negative cuproptosis-related genes (13). More and more novel cuproptosis-related genes (CRGs) have been identified in various tumors (15, 16). Evidence shows that lung cancer cells, including LUAD, also require glutamine to fulfill metabolic needs, which is important for the TCA cycle (17, 18). Numerous studies have developed different cuproptosis-related risk models to predict prognosis and immune infiltration in LUAD using bioinformatics analyses (16, 19–21). However, no studies of CRGs combined with stemness signatures in LUAD have been reported to date, and their roles in prognosis and immune landscape remain unknown.

Compared to conventional bulk sequencing, single-cell RNA sequencing (scRNA-seq) is capable of uncovering specific cell populations and intratumoral heterogeneity at the single-cell level (22, 23). Therefore, we for the first time identified the cuproptosis-related stemness genes (CRSGs) in LUAD by integrating bulk and scRNA-seq and constructed a prognostic signature to predict the prognosis, immune infiltration, stemness features, immunotherapy response, and drug sensitivity. Lastly, *in vitro* experiments were performed to investigate the expression and biological function of CRSGs. These findings highlight the essential role of CRSGs in LUAD patients, which might provide new insights into elucidating heterogeneity and developing more effective therapeutic targets for CSCs.

## Materials and methods

### Data acquisition and preprocessing

The Gene Expression Omnibus (GEO) database (https://www.ncbi.nlm.nih.gov/geo/) was used to analyze the scRNA-seq data of 11 LUAD samples (GSE131907 (24)). The bulk RNA-seq data of 541 LUAD samples and 59 para-carcinoma samples were obtained from the Cancer Genome Atlas (TCGA) database, including 491 patients with clinicopathologic and survival information (**Table S1**). Additionally, transcriptomic data from 19 LUAD samples is included in GSE141569 (25) as the external validation set. All the datasets were normalized by the limma package (26) and the R package (27). Simultaneously, in total of 10 cuproptosis marker genes and 2916 cancer stemness genes were obtained by literature review (13, 28) and related databases (29, 30).

### Single-cell data analysis and intercellular communication

The quality control of scRNA-seq data was performed using the Seurat R package (version 4.1.0) (31) to optimally eliminate potential doublets. Using Uniform Manifold Approximation and Projection (UMAP), the top 30 components from principal component analysis (PCA) on highly variable genes were chosen. Cells were clustered using the FindClusters function. The FindMarkers function was used to annotate cell types based on reported cell-specific marker genes (**Table S2**). The R package CellChat (version 1.1.3) (32) was used to evaluate cell–cell interactions based on the CellChatDB databases.

### The scores of stemness and cuproptosis at the single-cell level

To obtain the stemness signature gene set of LUAD, we downloaded 2916 stemness-related genes from the literature and database, and aligned them with single-cell genes. The scores of stemness signature were divided by median values, which were calculated by the AddModuleScore function in Seurat. The scores of cuproptosis for each cell were obtained by calculating the Area Under the Curve (AUC) value of key CRGs using the AUCell R package (version 1.18.1) (31). The UMAP embedding is colored by the AUC scores. The scores of cuproptosis signature were divided by the activity of cell clusters in LUAD scRNA-seq.

### Analysis of DEGs and cuproptosis-related molecular subtypes

DEGs were identified based on the TCGA-LUAD data by using the R package. DEGs were defined as |log2 FC|>2 with adjusted p<0.05 and visualized using heatmaps (33) and volcano plots from the R packages ggplot2 (34).

The consensus clustering analysis was used to identify different subtypes in LUAD based on cuproptosis-related DEGs by the “ConsensusClusterPlus” R package (35). To ascertain the K value, a cumulative distribution function (CDF) curve was employed, and the classification was verified by PCA in LUAD.

### Functional enrichment and gene set variation analysis

Using the clusterProfiler (36) package, the Gene Ontology (GO) (37) and Kyoto Encyclopedia of Genes and Genomes (KEGG) (38) enrichment analyses of the DEGs were performed. The dataset of immune cells was downloaded from TISIDB (39) (http://cis.hku.hk/TISIDB/download.php) using the GSVA package (40). The stemness and immune scores based on the gene expression matrix were calculated using the single sample gene set enrichment analysis (ssGSEA).

### Construction of the prognostic model and nomogram

Forest plots were drawn based on the results of univariate and multivariate Cox regression. By using univariate and least absolute shrinkage operator (LASSO) cox regression, a prognostic model based on differently expressed CRSGs was built. Cox regression coefficients using the formula:


$$\text{RiskScore'}=\sum \exp_{\text{Genei}}\times {\text{coef}}_{\text{Genei}}$$

Kaplan‐Meier (K-M) analysis and the receiver operator characteristic (ROC) curve were performed to estimate the OS using the R ‘survival’ and ‘timeROC’ packages. A nomogram for predicting the OS was built by using the rms R package. To assess the clinical value of nomograms, decision curve analysis (DCA) and clinical impact curves were used.

### Correlation analysis of immune infiltrating cells

The gene expression matrix of infiltrating immune cells was obtained by CIBERSORT (41) using the LM22 signature. The correlation of 22 immune cells was shown in a heatmap by the corrplot algorithm, and the correlation between immune infiltration and prognosis was calculated by the ggplot2 package. We also analyzed the correlation of prognostic genes with immune checkpoints.

### Anticancer drug sensitivity analysis

The anticancer drug sensitivity and markers of drug response were collected from the Genomics of Drug Sensitivity in Cancer (GDSC) database (42). A ridge regression model was built using gene expression profiles by the pRRophetic algorithm (43). The sensitivity of an anticancer drug was classified by IC50 values.

### Cell culture and transfection

The LUAD cell lines (A549 and SPC-A1) and human bronchial epithelial cells (BEAS-2B) were purchased from the Cell Bank of the Chinese Academy of Sciences (Shanghai, China). All cells were cultured in DMEM or RPMI-1640 medium (Hyclone, USA) supplemented with 10% fetal bovine serum (FBS; Gibco, USA).

The small interfering RNA of SPP1 (siSPP1) and control RNA (si-Ctrl) were synthesized by GenePharma Inc. (Shanghai, China). Lipofectamine 3000 (Invitrogen, USA) was used to transiently transfect the siRNA into cells. The sequence of siSPP1#1 is UAUUUUGGCCUUUAUUCUGUU, siSPP1#2 is GAGAATTGCAGTGATTTGCTTTT, and siSPP1#3 is AGGAAAAGCAGCTTTACAAAA. After 48 hours of incubation, the interfering effect was confirmed by Western blotting. The following antibodies were used: anti-SPP1 (ab302942, 1:1000, Abcam, USA), β-actin (ab8226, 1:1000, Abcam, USA).

### Quantitative real-time PCR

TRIzol reagent (Invitrogen, USA) was used to extract total RNA from the cells, and the cDNA synthesis kit (Takara, Japan) was used to reverse-transcribe the extracted total RNA into cDNA in accordance with the kit’s instructions. SYBR Green RT-PCR Kits (Takara, Japan) were used for the qPCR, and 2^−ΔΔCt^ was used to determine the relative mRNA expression. β-actin provided internal control. **Table 1** contained a list of the primers.

**Table 1:** Primer sequences used for qRT-PCR.

Gene	Primers	Sequence (5′–3′)
CDKN2A	Forward	GGAGGCCGATCCAGGTCAT
	Reverse	CACCAGCGTGTCCAGGAAG
GLS	Forward	CACTCAAATCAGGATTGCG
	Reverse	CCAGACTGCTTTTTAGCACTTT
FDX1	Forward	CCTGGCTTGTTCAACCTGTCA
	Reverse	CCAACCGTGATCTGTCTGTTAGTC
PDHA1	Forward	CAGACCATCTCATCACAGCCTACC
	Reverse	CCTCCTTTCCCTTTAGCACAACCT
PDHB	Forward	GACACTCCCATATCAGAGATGG
	Reverse	CTTGGCAGCTGAGTTTATAACC
DLD	Forward	GCCGACGACCCTTTACTAAGAAT
	Reverse	GGACCAGCAACTACATCACCAAT
KLF4	Forward	AACCTATACGAAGAGTTCTCAT
	Reverse	CCAGTCACAGTGGTAAGG
SCGB3A1	Forward	ATGTCCCCACAATCAGCAAG
	Reverse	CTCTGCAGCTGGAGCAAGG
COL1A1	Forward	GCTCCTCTTAGGGGCCACT
	Reverse	CCACGTCTCACCATTGGGG
SPP1	Forward	CAAATACCCAGATGCTGTGGC
	Reverse	TGGTCATGGCTTTCGTTGGA
C4BPA	Forward	CTACGCATACGGCTTTTCTGT
	Reverse	CCCATGTGAAACATCTGGCTTG
TSPAN7	Forward	CTCATCGGAACTGGCACCACTA
	Reverse	CCTGAAATGCCAGCTACGAGCT
CAV2	Forward	CGTGCCTAATGGTTCTGCCT
	Reverse	CGCTCGTACACACAATGGAGCA
CTHRC1	Forward	ATAATGGAATGTGCTTACAAGG
	Reverse	TTCCCAAGATCTATGCCATAAT
β-actin	Forward	CTTCGCGGGCGACGAT
	Reverse	CCACATAGGAATCCTTCTGACC

### Cell proliferation and migration assay

Cell proliferation was evaluated using the CCK-8 assay. The transfected SPC-A1 cells were seeded onto 96-well plates with 2×10^3^ cells/well and incubated for 5 days. Cell Counting Kit-8 (CCK-8) (Beyotime, China) was added and detected the absorbance of the solution at 490 nm. Transwell test was used to measure cell migration. Cells (2×10^5^ cells/ml) were added to the upper 24-well plate chamber with FBS-free medium, while the lower chamber was contained with 20% FBS medium. After 24 hours, the cells in the lower chamber were stained and counted under the 200x microscope.

### Tumorsphere formation assay

SPC-A1 cells (3×10^3^/well) were plated into an ultralow attachment 6‐well plate (Corning, USA) and incubated for 5-7 days. Serum-free DMEM/F12 (Gibco, USA) supplemented with 20 ng/mL epidermal growth factor (Sigma, USA), 20 ng/mL basic fibroblast growth factor (Sigma, USA), 20 μL/mL B27 (Invitrogen, USA), and 5 μg/mL insulin (Invitrogen, USA) was used to culture the cells. Morphology of CSC spheres was photographed under the 400x microscope.

### Statistical analysis

Using R programming (version 4.1.0), all statistical analyses were carried out. T-tests or the Mann-Whitney U test were used to compare continuous variables between groups. All p values were two-sided, and significance was indicated by *p* < 0.05.

## Results

### Clustering and differential analysis of scRNA-seq data

**Figure 1:**
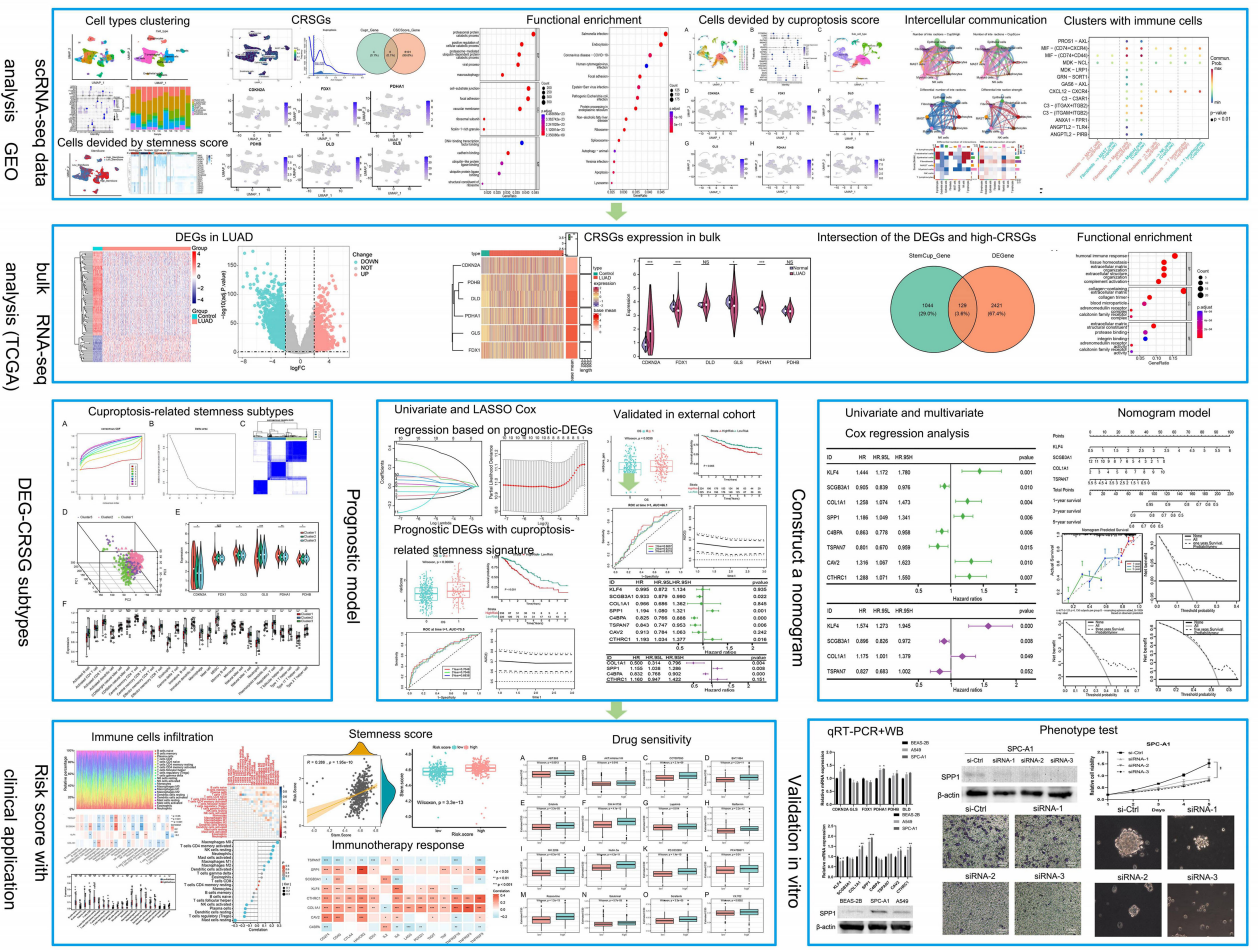
Flow chat in the study.

**Figure 2:**
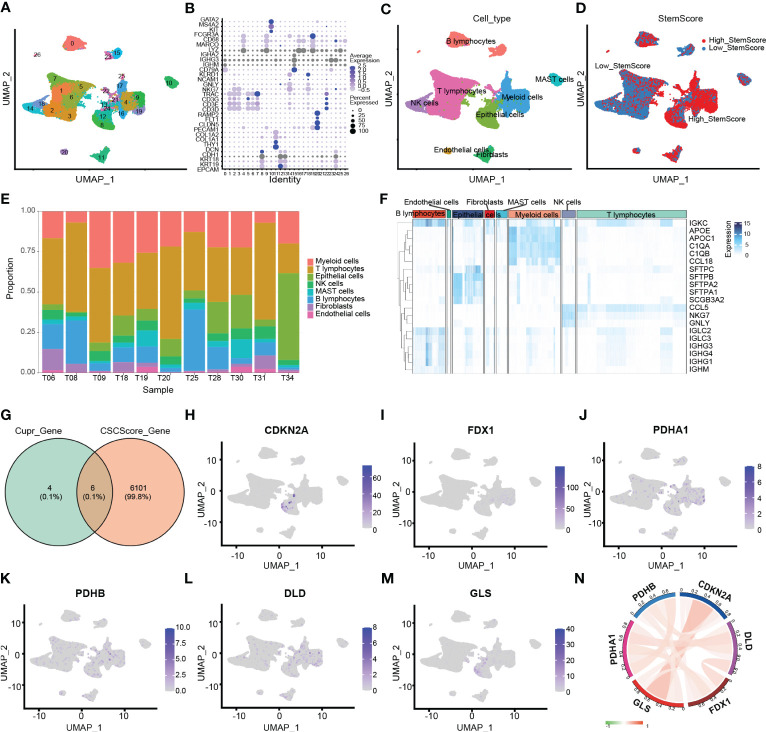
Clustering and differential analysis of scRNA-seq data. **(A)** Cells in scRNA-seq (GSE131907) were classified into 27 clusters by dimensional reduction and clustering analysis. **(B)** Marker gene expression in each cluster. **(C)** The UMAP diagram shows the distribution of the 8 major cell types in each sample. **(D)** The major cell types were divided into high- and low- stemness cells by the stemness score. **(E)** Histogram overlays display the proportion of cell types in each sample. **(F)** A heatmap showing the top 20 differentially expressed stemness genes in each cell type. **(G)** Venn diagram shows the intersection of differential stemness genes and cuproptosis-related genes. **(H-M)** Expression of CRSGs in different cell types: CDKN2A, FDX1, PDHA1, PDHB, DLD, and GLS. **(N)** The circle plot shows the correlation between CRSGs.

The flow chat was shown in **Figure 1**. After quality control, we used scRNA-seq data (GSE131907) to obtain gene expression profiles for 45,149 cells from 11 primary LUAD samples. As shown in **Figure 2A**, these cells were classified into 27 clusters by the KNN algorithm. Subsequently, clusters were annotated into 8 major cell types (**Figures 2B**; **S1A**) based on the expression of marker genes (**Table S2**). They were epithelial cells (contain non-malignant cells and cancer cells), myeloid cells, T lymphocytes, natural killer (NK) cells, B lymphocytes, fibroblasts, mast cells, and endothelial cells (**Figure 2C**). There is a relatively high proportion of T lymphocytes and a low proportion of endothelial cells (**Figure 2E**). Then, we divided each cell into high- and low- stemness cells according to the median value of the stemness score (**Figure 2D**). Furthermore, a total of 6107 differentially expressed stemness genes were identified, and the top 20 genes were shown in the heatmap (**Figure 2F**).

### Analysis of cuproptosis score based on stemness signature and functional enrichment

**Figure 3:**
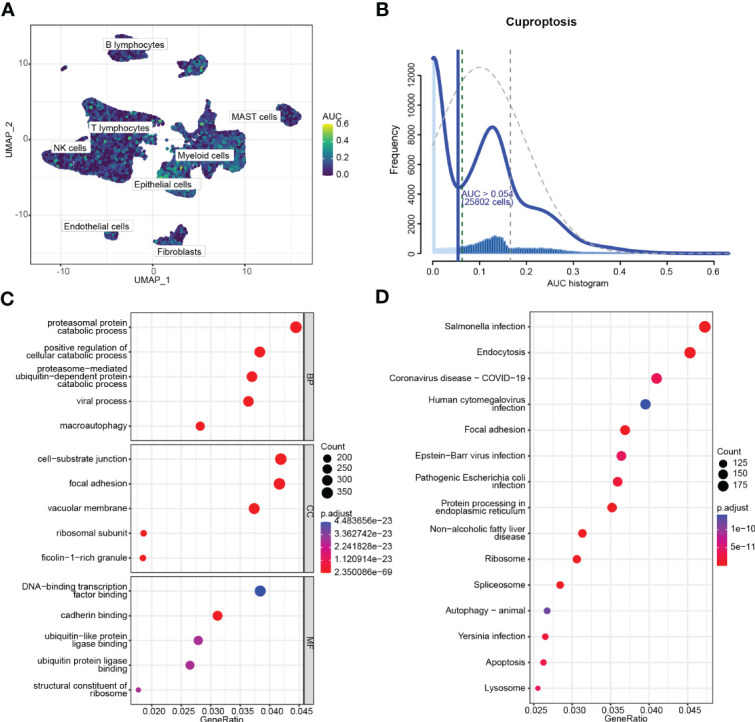
Analysis of cuproptosis score based on stemness signature and functional enrichment in scRNA-seq. **(A)** The UMAP plot shows the cuproptosis score based on stemness signature in each cell type. **(B)** The distribution graph of AUC. High-cuproptosis score cells were selected using AUCell function by AUC>0.054. **(C)** GO enrichment analysis of DEGs between the high- and low- cuproptosis score cells based on stemness signature. **(D)** KEGG pathway analysis of DEGs.

Through the intersection of the 6107 differentially expressed stemness genes and 10 cuproptosis-related genes, 6 CRSGs (CDKN2A, GLS, FDX1, PDHA1, PDHB, and DLD) were obtained (**Figure 2G**). We further explored that they were mainly expressed in epithelial (contain non-malignant cells and cancer cells), myeloid cells and T lymphocytes by scRNA-seq. (**Figures 2H–M**). Additionally, there was a positive correlation among these CRSGs, the expression of CDKN2A was positively correlated with GLS (cor = 0.394) (**Figure 2N**). These genes were significantly more active in epithelial and myeloid cells (**Figure 3A**). In total, 25802 cells with a high-cuproptosis score based on stemness signature were screened by the AUCell R package (AUC > 0.054) (**Figure 3B**).

We further explored the functional enrichment between the high- and low- cuproptosis score cells based on stemness signature by GO and KEGG analyses. They were most enriched in the metabolic microenvironments and cancer-related pathways, such as protein catabolism, DNA-binding proteins, and endocytosis (**Figure 3C, D**; **Table S3**-**4**).

### Clustering subtypes of high-cuproptosis score with stemness signature in single-cell data

**Figure 4:**
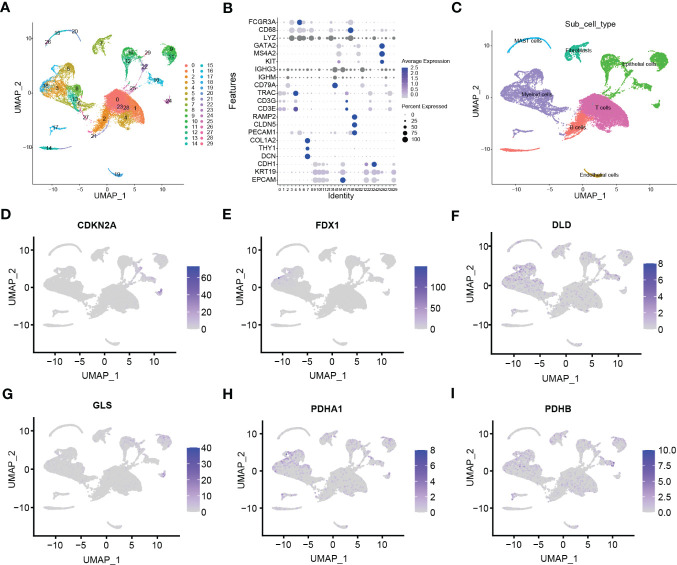
Clustering subtypes of high-cuproptosis score with stemness signatures in single-cell data. **(A)** The UMAP diagram shows the high-cuproptosis score stemness cells were classified into 30 clusters using dimensional reduction and clustering analysis. **(B)** Marker gene expression in each cluster. The bigger the dots, the higher the cell proportion. **(C)** Seven recognized cell types based on previous cell markers. **(D-I)** Expression of CRSGs in each cell type: CDKN2A **(D)**, FDX1 **(E)**, DLD **(F)**, GLS **(G)**, PDHA1 **(H)**, PDHB **(I)**.

After obtaining the high-cuproptosis score stemness cells, we classified them into 30 clusters by the KNN algorithm (**Figure 4A**). Finally, cell types were recognized based on previous cell markers (**Figures 4B**; **S1B**): epithelial cells (contain non-malignant cells and cancer cells), myeloid cells, T lymphocytes, fibroblasts, B lymphocytes, mast cells, and endothelial cells (**Figure 4C**). Cell clusters were almost consistent with the distribution by stemness score above. Additionally, the expression of CRSGs in subtypes was similar to previous results from scRNA-seq (**Figures 4D–I**).

### Intercellular communication between cuproptosis stemness cluster and others

**Figure 5:**
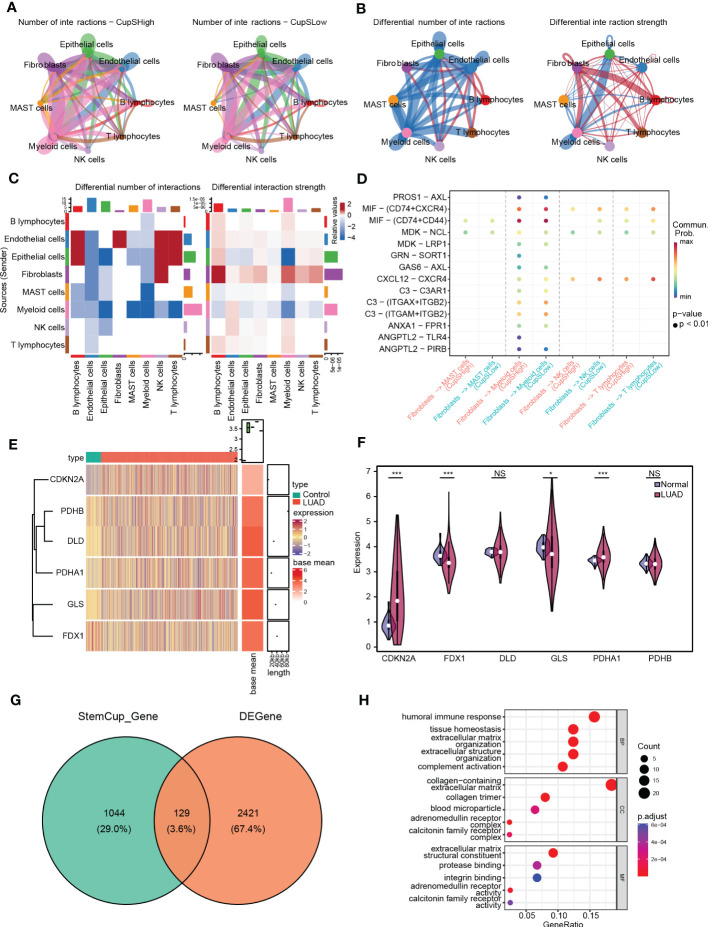
Intercellular communication between cuproptosis score stemness cluster and others. **(A)** Cell-cell communication network of high-(Left) and low- (Right) cuproptosis score stemness cluster with others by CellChat. **(B)** Differential interaction number (Left) and strength (Right) between the cuproptosis score stemness cluster with others. **(C)** The heatmap shows the differential interaction number and strength. **(D)** Ligand-receptor interactions plot. **(E)** The heatmap shows the differential expression of CRSGs in bulk RNA-seq. **(F)** Violin plots showing the expression of CRSGs between LUAD and para-carcinoma tissues in TCGA dataset. **(G)** Venn diagram shows the intersection of the DEGs and the marker genes in high-cuproptosis score stemness clusters. **(H)** GO analysis of the intersecting genes. *p* values were shown as: *, *p*<0.05; **, *p*<0.01; ***, *p*<0.001; NS, no significance.

CellChat was used to delineate intricate a cell-cell network from scRNA-seq. **Figure 5A** shows the intercellular communication of high- and low- cuproptosis score stemness cluster that mainly occurred in epithelial, endothelial, fibroblast, lymphocytes, and myeloid cells with differential interaction numbers and strengths. Further analysis suggested that high-cluster was more associated with immune cells, such as NK cells and lymphocytes, and less associated with epithelial cells, endothelial cells and myeloid cells (**Figures 5B, C**). Moreover, ligand-receptor pair analysis revealed that fibroblasts preferred to communicate with immunocytes through MIF-(CD74+CXCR4), MIF-(CD74+CD44) and MDK-NCL (**Figure 5D**).

### Characteristics of CRSGs in the bulk RNA-seq of LUAD

Further, we examined 2550 DEGs in total, including 985 genes upregulated and 1565 genes downregulated in TCGA-LUAD (**Figures S1C-D**). The expression of CRSGs in bulk RNA-seq showed that CDKN2A and PDHA1 were higher in LUAD (p < 0.001), while FDX1 and GLS were lower in LUAD (p < 0.05), and with unaltered levels of DLD and PDHB (p > 0.05) (**Figures 5E, F**). Additionally, through the intersection of the DEGs and the marker genes in high-cuproptosis stemness cluster, a total of 129 genes were obtained (**Figure 5G**; **Table S5**). GO analysis showed they were mostly related to immune features and complement activation. (**Figure 5H**; **Table S6**).

### Analysis of cuproptosis-related stemness subtypes and immune infiltration in LUAD

**Figure 6:**
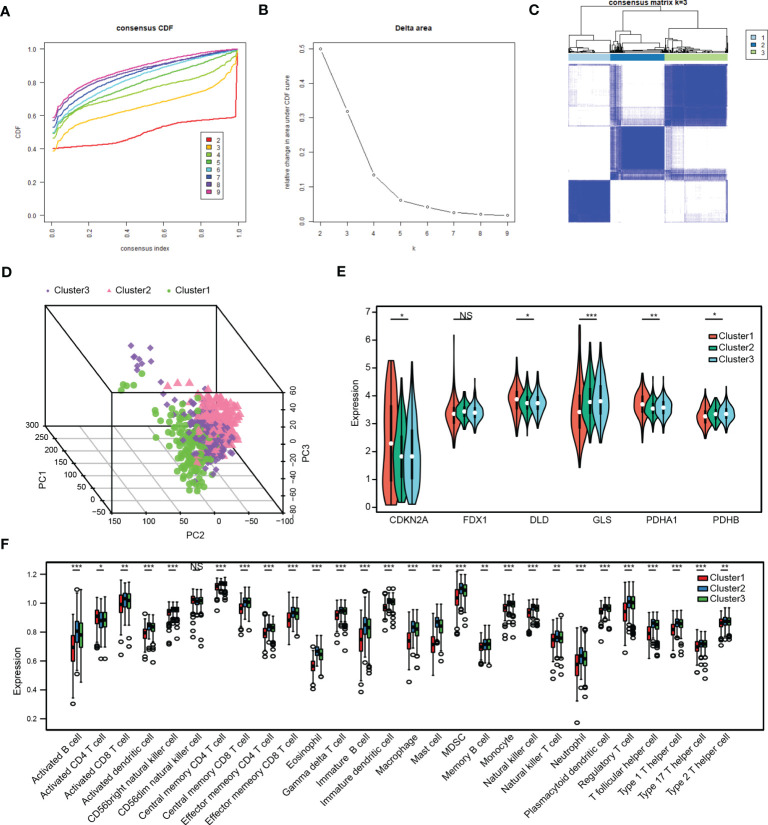
Analysis of cuproptosis-related stemness subtypes and immune infiltration in LUAD. **(A)** Plot of the Cumulative Distribution Function (CDF). **(B)** Delta area. **(C)** Unsupervised clustering heatmap when k=3. **(D)** Three distinct cuproptosis-related stemness subtypes were identified based on intersecting DEGs by principal component analysis (PCA). **(E)** Expression of CRSGs in the three clusters. **(F)** A box plot displaying the differences in immune cells that have infiltrated the three clusters by ssGSEA analysis. *, *p*<0.05, **, *p*<0.01, ***, *p*<0.001, NS, no significance.

Three distinct cuproptosis-related stemness subtypes were identified (Cluster 1-3) based on 129 intersecting DEGs by unsupervised clustering. (**Figures 6A–C**). The clustering criteria were k=3, and the results were confirmed by PCA (**Figure 6D**, **Figure S1E**). Furthermore, most CRSGs except FDX1, were significantly differentially expressed among the three clusters (p < 0.05) (**Figure 6E**).

Next, the immune infiltration score of the 28 immune cell types was evaluated in the three subtypes by employing the ssGSEA analysis (**Figure 6F**). The results showed that most immune infiltrating cells like activated B cells, CD4+ T cells, CD8+ T cells, myeloid-derived suppressor cells (MDSCs), and NK cells were significantly lower in Cluster 1, indicating that patients in Cluster 1 would be more insensitive to immunotherapy.

### Construction and validation of the prognostic model with cuproptosis-related stemness signature

**Figure 7:**
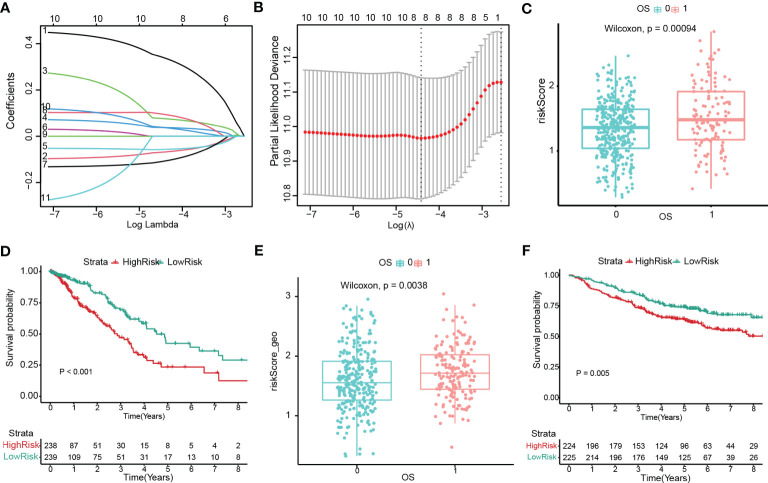
Construction and validation of the prognostic model with cuproptosis-related stemness signature. **(A, B)** Eight prognostic CRSGs were filtered to construct a prognostic model by LASSO-Cox regression. **(C)** The risk score for patients was validated in an internal cohort (TCGA-LUAD) and marked as low- (blue) and high-risk (red) (p=0.00004). **(D)** Kaplan-Meier survival analysis the survival probability between the low- and high-risk groups in TCGA-LUAD (p<0.001). **(E)** The risk score for patients was validated in an external cohort (GSE141569) and marked as low- and high-risk (p=0.0038). **(F)** K–M survival analysis of the risk score in an external cohort (p=0.005).

A prognostic signature was constructed by univariate and LASSO Cox regression to select the most significantly prognostic CRSGs among the 129 DEGs (**Figures 7A, B**). As a result, eight genes (KLF4, SCGB3A1, COL1A1, SPP1, C4BPA, TSPAN7, CAV2, and CTHRC1) with minimal lambda (p = 0.01) were finally screened out to construct the prognostic model. Internal validation cohort (TCGA-LUAD) shows patients with a high-risk score exhibited a worse OS (p=0.00004, **Figure 7C**). Similarly, K-M analysis showed that patients in the high-risk group had significantly lower survival rates (p < 0.001, **Figure 7D**). The ROC curves for 1-, 2- and 3- year OS were calculated, with AUCs of 0.7049, 0.7049, and 0.6836, respectively (**Figures S2A, B**). Additionally, we also validated in external cohort (GSE141569). Consistent with the above results, patients with higher risk scores showed higher mortality (p = 0.0038, **Figure 7E**). The K-M curve and AUC values also exhibited higher OS rates in the low-risk group (p = 0.005, **Figurea 7F**; **S2C, D**). All the results indicated that the risk score may be a trustworthy and accurate model to predict the prognosis of LUAD.

### Construction of the nomogram for LUAD patients

**Figure 8:**
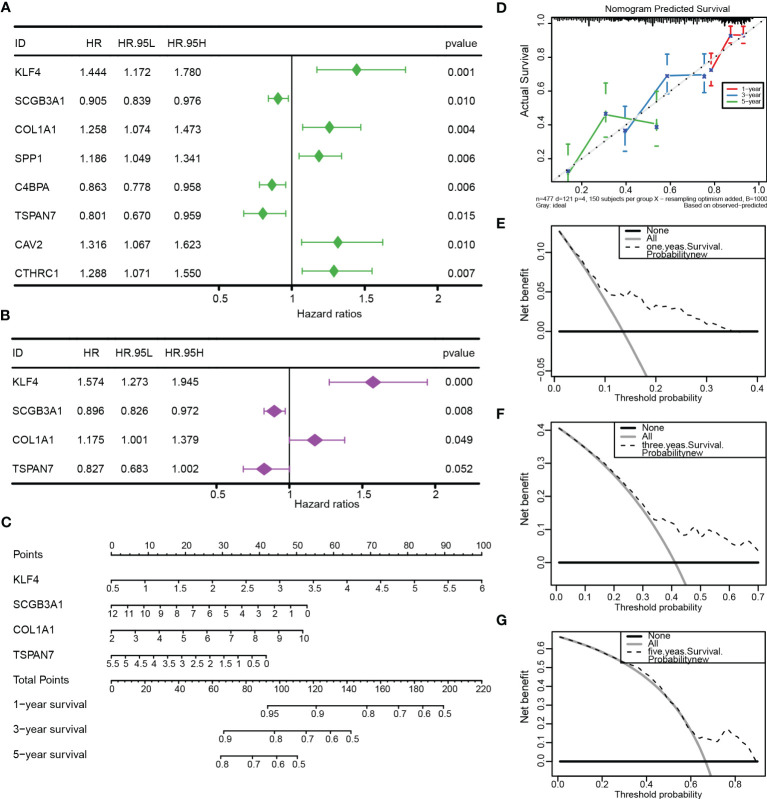
Construction and validation of the nomogram. **(A, B)** Univariate and multivariate Cox regression based on TCGA-LUAD. **(C)** The nomogram was constructed to predict OS. **(D)** The calibration curve demonstrated the validity and accuracy of the nomogram. **(E-G)** The decision curve analysis (DCA) for the nomogram at 1, 3, and 5 years.

To further apply the prognostic model, we performed the univariate and multivariate Cox regression analysis (**Figure 8A-B**; **Table S7**) based on the clinical information (**Table S1**) and CRSGs features from TCGA-LUAD. Similar results were validated in an external cohort (**Figures S3A, B**). The nomogram was constructed based on the results of multivariable Cox regression (**Figure 8C**). The accuracy of the nomogram’s 1-, 3-, and 5-year survival predictions was demonstrated by calibration curves. (**Figure 8D**). Meanwhile, the DCA also indicated that LUAD patients were more likely to benefit from the nomogram model (**Figures 8E–G**).

### Immune infiltration profiles and stemness score based on a prognostic signature

**Figure 9:**
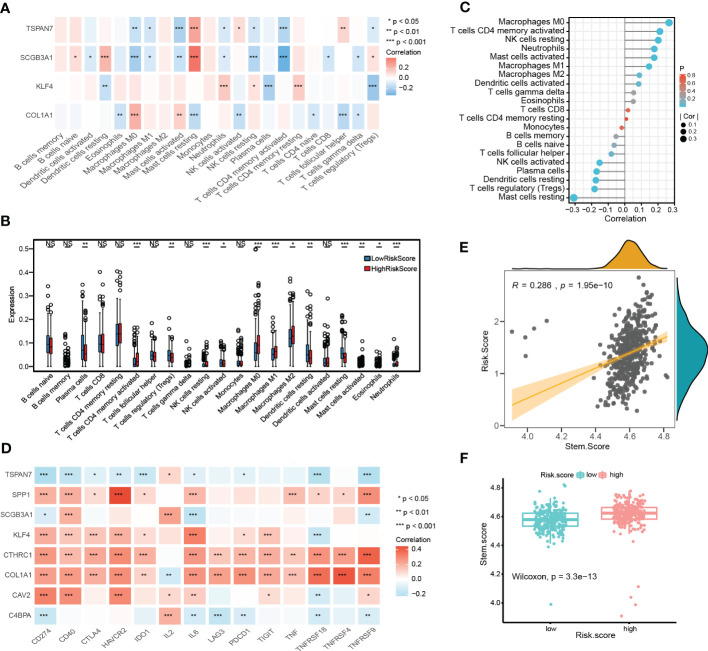
Immune landscape and stemness score based on prognostic signature. **(A)** The heatmap shows the correlation between CRSGs with prognostic signature and immune cells. **(B)** The differences in immune cells between high- and low- risk groups. **(C)** Lollipop plot showing the correlation between immune cells and the risk score. The size of the bubbles represents the strength of the correlation. **(D)** The correlation between prognostic CRSGs and immune checkpoint genes. Red, positive correlation; blue, negative correlation. **(E)** Correlation analysis between stemness score and the risk score (p=1.95e-10). **(F)** High- and low-risk groups’ stemness score were compared (p=3.3e-13). *p* values were shown as: *p<0.05; **p<0.01; ***p<0.001.

We further performed the CIBERSORT algorithm to assess the proportion and correlation of immune cells in each LUAD patient (**Figures S3C, D**). Correlation analyses found that CRSGs with prognostic signature were associated with most of the 22 immune cells (**Figure 9A** **).** Besides, there were significant immune cell differences between the high- and low-risk groups. (**Figure 9B**). Finally, a correlation analysis between risk score and immune infiltration was performed, which revealed that risk score was positively correlated with M0 macrophages, memory CD4+ T cells, and resting NK cells but negatively correlated with activated NK cells, resting mast cells and Tregs **(** **Figure 9C** **)**

Moreover, the stemness score was calculated using ssGSEA, and correlated with the risk score. A positive association was found between risk score and stemness score (r = 0.286, p = 1.95e-10, **Figure 9E**), which indicated that patients with a higher risk score also had a higher stemness score and more CSC features (p = 3.3e-13, **Figure 9F**).

### Immunotherapy response and drug sensitivity

To further evaluate the immunotherapy response with CRSGs in LUAD, a correlation analysis between the prognostic CRSGs and the immune checkpoint genes was conducted. KLF4, COL1A1, SPP1, CAV2, and CTHRC1 were positively related to the top 14 immune checkpoint genes, of which CTHRC1 and COL1A1 had the highest correlation, while TSPAN7, C4BPA, and PSMB9 showed a negative correlation (**Figure 9D**). Taken together, these results indicated that the prognostic CRSGs could be a useful biomarker to predict LUAD patients who will benefit from immunotherapy.

We also evaluated potential anti-tumor drugs between high- and low- risk group based on drug sensitivity profiles from the GDSC database. The top 16 sensitivity drugs were selected by calculating IC50 values, such as AKT-VIII, EHT-1864, GW-441756, erlotinib, lapatinib, etc., implying that patients in the high-risk group were more sensitive to chemotherapy and targeted therapy (**Figures S4A-P**).

### Validation of cuproptosis-related stemness signature in vitro

**Figure 10:**
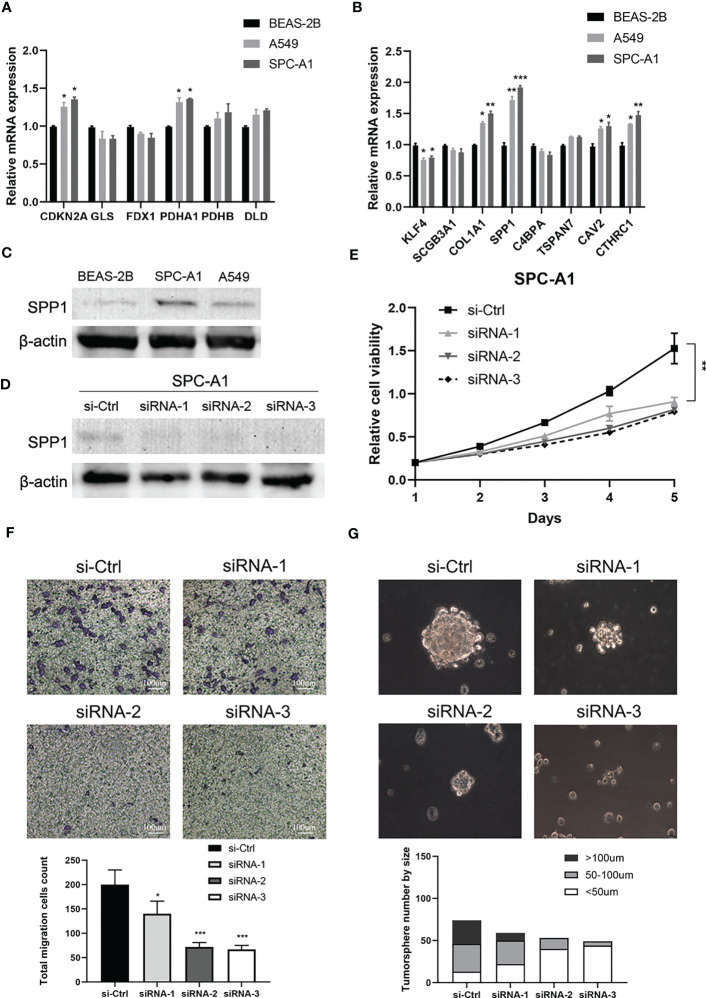
Validation of cuproptosis-related stemness signature in LUAD cells. **(A)** The mRNA expression of CRSGs in LUAD cells A549 and SPC-A1 and normal bronchial epithelial cells BEAS-2B was analyzed by qRT-PCR. **(B)** The mRNA expression level of DEGs with prognostic signature. **(C)** The protein expression of SPP1 in A549, SPC-A1, and BEAS-2B by Western blot. **(D)** Western blot assay verified the efficiency of SPP1 knockdown in SPC-A1. **(E)** CCK-8 assay was used to evaluate the effect of SPP1 on cell proliferation. **(F)** Transwell assay to assess the effect of SPP1 on the migration of SPC-A1 cells (scale bar, 100um). And the corresponding statistical plot was displayed. **(G)** Representative images show the effect of SPP1 knockdown on the tumorsphere formation ability of SPC-A1 cells, which were cultured in stemness medium for 7 days (scale bar, 100um). Quantitative analysis was counted by sphere diameters. *p* values were shown as: *p<0.05; **p<0.01; ***p<0.001.

Finally, we further verified the mRNA expression of CRSGs and DEGs with prognostic signature in LUAD cells. Compared with normal bronchial epithelial BEAS-2B, the expression of CDKN2A, PDHA1, COL1A1, SPP1, CAV2, and CTHRC1 was significantly upregulated in A549 and SPC-A1 as expected with the above analyses **(**p < 0.05, **Figure 10A, B**). SPP1 in particular was found to be highly expressed at both the mRNA and protein levels **(**p < 0.001, **Figure 10C**). Thus, SPP1 was selected to further explore biological function *in vitro*. The effectiveness of SPP1 silencing was confirmed by western blot (**Figure 10D**). The CCK-8 and transwell assays revealed that the knockdown of SPP1 significantly suppressed the proliferation and migration of LUAD cells **(**p < 0.01, **Figures 10E, F**). Furthermore, tumorsphere numbers and sizes were markedly reduced in SPC-A1 after transfection with siRNA-2 and -3, indicating that SPP1 promoted cancer stemness and might be a potential target for CSCs (**Figure 10G**). Together, these results strongly support the reliability of our bioinformatics analysis.

## Discussion

LUAD accounts for approximately 50% of all lung cancers, with a high morbidity and mortality rate due to its properties of high metastasis, radio-chemotherapy resistance, and immunotherapy insensitivity (1, 2). CSCs, only a small population of cancer cells possess the stemness abilities of tumor-initiation, self-renewal, and unlimited proliferation, which are considered the “root” of LUAD recurrence, metastasis, and resistance (4, 5). Thus, there is an urgent need to identify more effective therapeutic strategies for CSCs.

Low levels of ROS are essential to maintaining stemness in CSCs (44). A promising new approach for generating intracellular ROS and mitochondrial oxidative stress by copper ionophores has emerged, with an intrinsic selectivity for CSCs of the lung, colorectal, and breast (8, 11, 12). Copper acts as a “double-edged sword” and plays an essential role in cancer development, metastasis, and immunomodulatory (9, 10). In fact, a novel form of copper-dependent cell death that is triggered by copper ionophores, called cuproptosis, is accompanied by the accumulation of ROS and mitochondrial metabolism (13, 14). Previous studies have identified several genes and lncRNAs related to cuproptosis in LUAD (20, 21, 45–47), and developed a cuproptosis signature that correlates with the prognosis and tumor microenvironment of LUAD patients (16, 19, 48). Therefore, cuproptosis may play an important role in LUAD and provide new insights into the treatment of CSCs.

To the best of our knowledge, no studies of cuproptosis-related genes combined with the stemness signature in LUAD have been reported, and their roles in prognosis and the immune landscape remain unknown. Due to the high heterogeneity of CSCs (49, 50), we first systematically analyzed the CRSGs in LUAD by integrating bulk and single-cell RNA-seq. A total of 6 CRSGs were screened out, including CDKN2A, GLS, FDX1, PDHA1, PDHB, and DLD; most of them have been reported in the direct regulation of cuproptosis and cancer progression (13). In our study, the expression of CRSGs in bulk RNA-seq showed that CDKN2A and PDHA1 were significantly higher, while FDX1 and GLS were lower in LUAD patients, and with unaltered levels of DLD and PDHB. Although CDKN2A showed a high mutation frequency in various cancers, the expression of CDKN2A was overexpressed in many tumors and associated with immunosuppression and poor prognosis (51). CDKN2A genomic alterations were associated with urothelial carcinoma treated with immune checkpoint inhibitors (ICIs) (52). PDHA1 is crucial to metabolic reprogramming and is often aberrantly expressed in various tumors (53). In LUAD, patients with high expression of PDHA1 had a significantly negative correlation with poor prognosis and immune infiltration (54). Our further qRT-PCR assay validated the expression trend in the datasets, with only CDKN2A and PDHA1 having statistically significant differences (p < 0.05), which may be attributed to the differences between tissues and cell lines.

Based on the expression of 129 intersecting DEGs in LUAD, cells were classified into three cuproptosis-related stemness subtypes (Cluster 1-3) by unsupervised clustering. Additionally, functional enrichment analysis showed that those subtypes were enriched in cancer and immune-related pathways. Thus, we further explored the association between the subtypes and immune infiltration. Notably, most of the immune infiltrating cells like activated B cells, CD4+ T cells, CD8+ T cells, MDSCs, and NK cells were significantly lower in Cluster 1, indicating that patients in Cluster 1 would be insensitive to immune treatment (55). Furthermore, we used CellChat to delineate intercellular communication at the single-cell level, and a high-cluster had more communication with immune cells such as fibroblasts, NK cells, T lymphocytes, and B lymphocytes than a low-cluster. Further potential ligand-receptor interactions including MIF-(CD74+CXCR4), MIF-(CD74+CD44) and MDK-NCL have also been found (56). The persistent upregulation of CD74 could impair MHC class II antigen presentation, contributing to immune escape and promoting tumor metastasis (57). Overall, cuproptosis might bridge cancer stem cells and immunocyte infiltration to affect LUAD progression.

More importantly, to quantify the prognosis of cuproptosis-related stemness signature in each LUAD patient, we constructed a risk score based on the 129 intersecting DEGs by LASSO and univariate regression. Then, 8 prognostic genes with cuproptosis-related stemness signature (KLF4, SCGB3A1, COL1A1, SPP1, C4BPA, TSPAN7, CAV2, and CTHRC1) were involved in the novel prognostic model, which stratified LUAD patients into high- and low-risk groups. The K-M survival and ROC curves, as expected, showed that patients in the high-risk group had a poor overall survival (OS), which was validated in both the TCGA internal cohort and the GSE 141569 external cohort. By combining the risk signature with clinical information, a more accurate nomogram was constructed to predict the OS of LUAD patients. All the results indicated that cuproptosis-related stemness signature could serve as a solid predictive model for LUAD.

Among the eight CRSGs with prognostic signature identified in this study, COL1A1, SPP1, CAV2 and CTHRC1 were significantly upregulated in A549 and SPC-A1, while KLF4 was downregulated in LUAD cells. SPP1 in particular was found to be highly expressed at both the mRNA and protein levels (p < 0.001). Secreted phosphoprotein 1 (SPP1), also called osteopontin, has been demonstrated overexpressed in many cancers including LUAD and was correlated with a poor OS (58). SPP1 can induces EMT through the PI3K/Akt and MAPK/ERK1/2 pathways in lung cancer (59). It can enhance EGFR-TKI resistance by up-regulating integrin αVβ3 (60) and promote colorectal cancer stem cell-like properties by PI3K/AKT/GSK3 (61). Knockdown of SPP1 greatly decreased stemness features in cancer-associated fibroblasts treated with pancreatic cancer cells (62). Moreover, SPP1 was also considered as a cuproptosis-related gene in similar research based on database and learning algorithm (63). Our further *in vitro* experiments revealed that the silencing of SPP1 inhibited the proliferation, migration, and stemness sphere-forming capacities of LUAD cells. Therefore, SPP1 might serve as a novel therapeutic target for lung CSCs. Nevertheless, more research is needed to unravel the underlying mechanism of SPP1 to regulate cuproptosis in LUAD.

Besides, we also analyzed the correlation between the prognostic signature and the immune landscape and stemness score in each LUAD patient. The results revealed that the risk score was significantly correlated with correlated with immune cell infiltration. The high-risk group has more resting NK cells and less activated NK cells. We did not observe a significant difference in CD8+ T cells between risk scores and prognosis may be related to the immune escape. A positive relationship was discovered between risk score and stemness score, indicating that patients with a higher risk score had more stemness features. Moreover, the predictive effect of the CRSGs with prognostic signature for immunotherapy was also evaluated. In our study, KLF4, COL1A1, SPP1, CAV2, and CTHRC1 had a high positive relationship with the immune checkpoint genes, while TSPAN7, C4BPA, and PSMB9 showed a negative correlation. Patients in the high-risk group were more susceptible to chemotherapy and targeted therapy based on drug sensitivity analysis. Taken together, we speculated that our model was capable of reflecting the immune infiltration and immunotherapy in LUAD.

Nowadays, increasing studies of CRGs, lncRNAs, and their prognostic value for lung cancer have been published. We for the first time identified the CRGs combined with stemness signature by integrating bulk and sc-RNAseq, and the prognosis and immune landscape in LUAD were also investigated. Inevitably, there were several limitations in this study. First, our research was mainly based on public databases and was retrospective, though we have validated the prognostic signature in internal and external cohorts, and further validations using prospective multi-center studies are needed. Moreover, although we have verified the expression of CRSGs and the functional roles of target gene by cellular assays, the underlying cuproptosis mechanism of CRSGs in LUAD needs to be further investigated, and more studies directly connected to cuproptosis features of SPP1 (such as the elesclomol concentration in different LUAD cell lines and the intensity of intracellular cuproptosis at different expression levels of SPP1) *in vitro* are required.

## Conclusion

Taken together, we comprehensively identified the CRSGs in LUAD and constructed a risk signature based on differentially expressed CRSGs, which was closely associated with the prognosis, immune infiltration, immunotherapy response, stemness features, and drug sensitivity. Additionally, the expression and biological function of CRSGs were also evaluated *in vitro*. These findings highlight the clinical significance of CRSGs in LUAD patients, and provide new insights for developing more effective therapeutic targets for lung CSCs in the future.

## Data availability statement

The datasets presented in this study can be found in online repositories. The names of the repository/repositories and accession number(s) can be found in the article/**Supplementary Material**.

## Author contributions

JY and ZW designed the study; HD and KL collected data; LY and JQ analyzed the data; JY and HD wrote the manuscript draft; JY and ZW revised the manuscript; JQ performed the experiments. All authors contributed to the article and approved the submitted version.
