# Supplementary material for: Identification and validation of a novel cuproptosis-related stemness signature to predict prognosis and immune landscape in lung adenocarcinoma by integrating single-cell and bulk RNA-sequencing
Source: Front Immunol. 2023 May 23;14:1174762. doi: 10.3389/fimmu.2023.1174762 (PMC10242006; doi:10.3389/fimmu.2023.1174762)
Supplement: Supplementary file 1 [file DataSheet_1.docx]

**Supplementary figures**

**
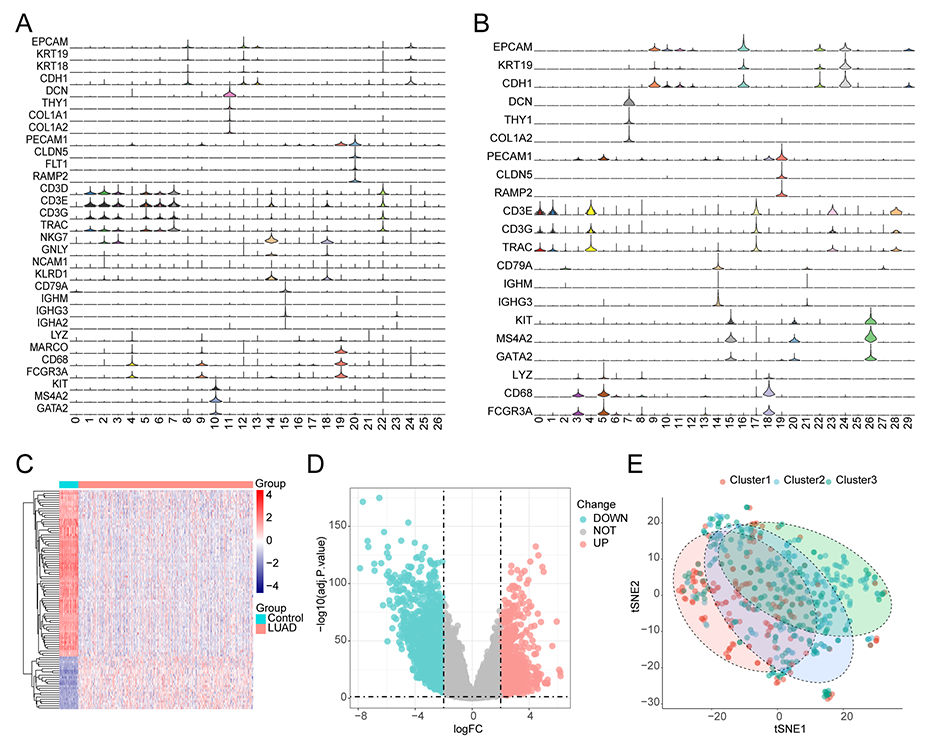
**

**Fig S1.** (A) Expression of marker genes in each cluster was represented by violin plots. (B) Violin plots shows the expression of marker genes in high-cuproptosis stemness clusters. (C-D) Differential expression between LUAD and para-carcinoma tissues in TCGA-LUAD by heatmap and volcano plot. Red represents high expression; blue represents low expression. (E) tSNE showing the three distinct clusters which were identified by consensus clustering (pale red, cluster1; blue, cluster2; green, cluster3).


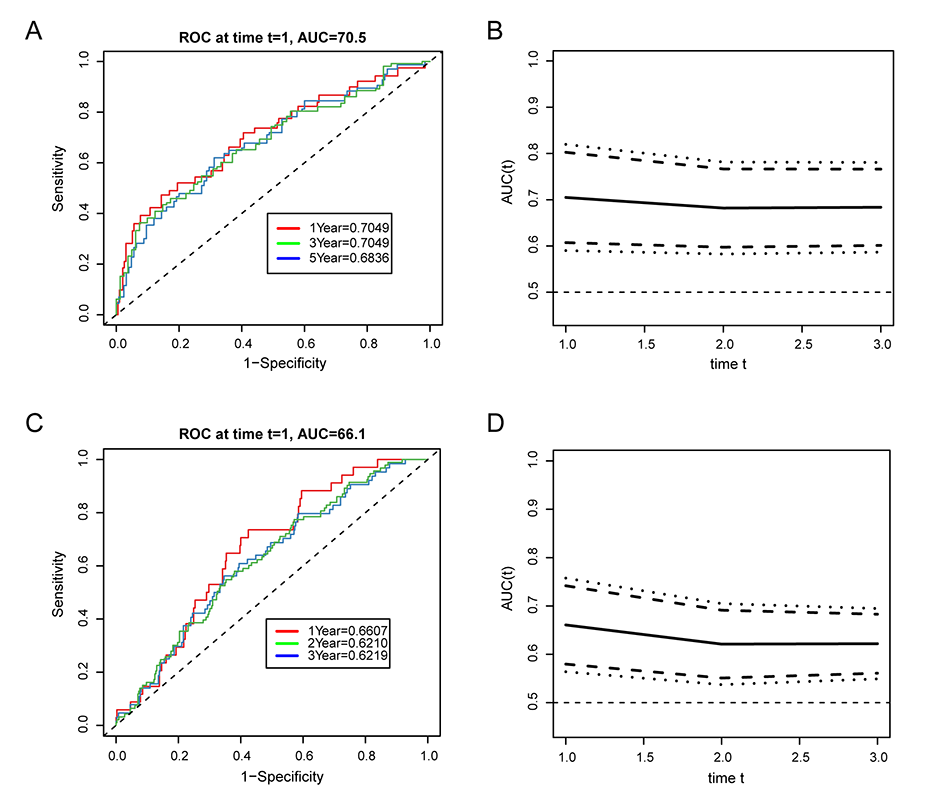


**Fig S2.** (A-B) ROC curves for 1-, 2-, and 3- year OS were calculated in TCGA-LUAD cohort, with AUCs of 0.7049, 0.7049 and 0.6836, respectively. (C-D) 1-, 2-, and 3-year ROC curves for the GSE141569 dataset, with AUCs of 0.6607, 0.6210 and 0.6219, respectively.


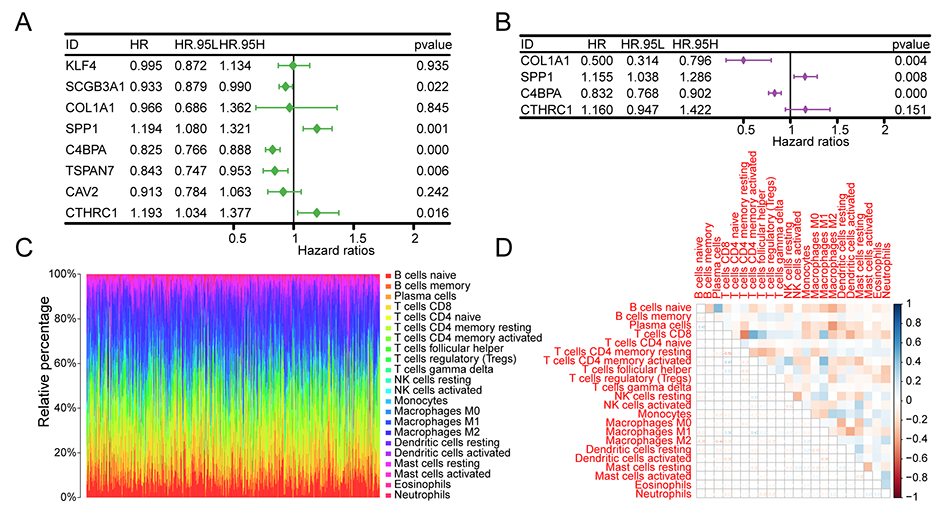


**Fig S3.** (A-B) Univariate and multivariate Cox regression were validated in external cohort. (C-D) Barplot and heatmap showing the proportion and correlation of the 22 immune cells in each TCGA-LUAD patient by using CIBERSORT.


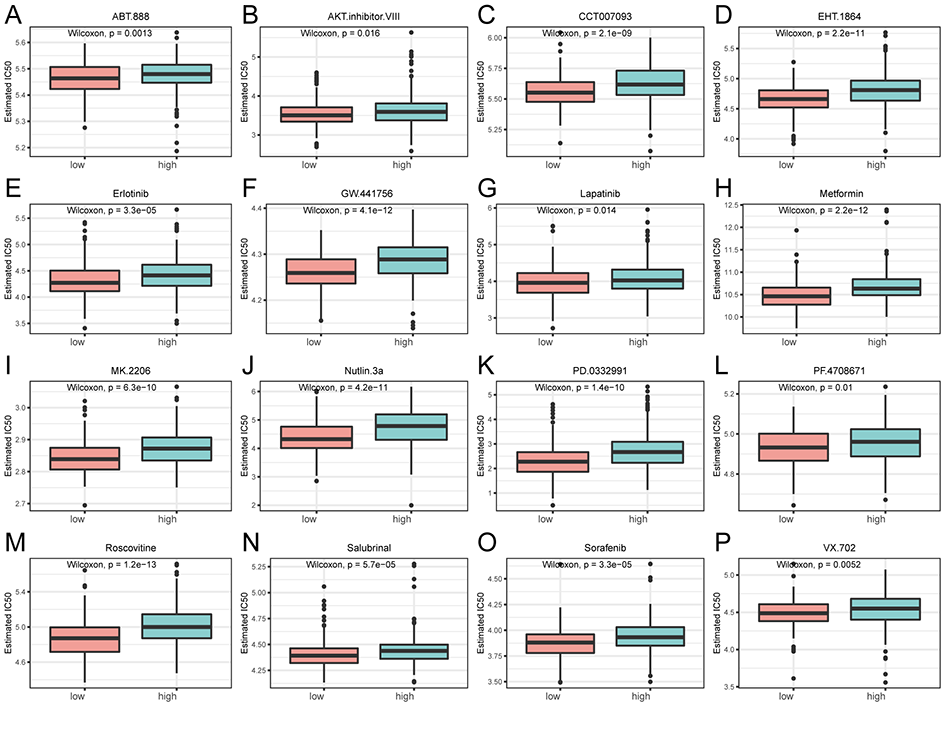


**Fig S4.** (A-P) Box plots showing the drug sensitivity between high- and low- risk groups. Red represents high-risk group, bule represents low-risk group.

**Table S1** Clinical information of the LUAD patients included in this study.

|  | Overall | Dead | Survive | p |
| --- | --- | --- | --- | --- |
| n | 491 | 121 | 370 |  |
| OS.time (mean (SD)) | 1.47 (2.27) | 2.33 (2.13) | 1.19 (2.25) | <0.001 |
| Age (mean (SD)) | 65.34 (10.03) | 66.19 (10.58) | 65.07 (9.85) | 0.286 |
| Gender = Female/Male (%) | 264/227 (53.8/46.2) | 67/54 (55.4/44.6) | 197/173 (53.2/46.8) | 0.762 |
| StageT (%) |  |  |  | <0.001 |
| T1 | 169 (34.4) | 25 (20.7) | 144 (38.9) |  |
| T2 | 257 (52.3) | 74 (61.2) | 183 (49.5) |  |
| T3 | 44 (9.0) | 11 (9.1) | 33 (8.9) |  |
| T4 | 18 (3.7) | 11 (9.1) | 7 (1.9) |  |
| TX | 3 (0.6) | 0 (0.0) | 3 (0.8) |  |
| StageN (%) | |  |  | <0.001 |
| N0 | 317 (64.6) | 52 (43.0) | 265 (71.6) |  |
| N1 | 92 (18.7) | 35 (28.9) | 57 (15.4) |  |
| N2 | 70 (14.3) | 32 (26.4) | 38 (10.3) |  |
| N3 | 2 (0.4) | 0 (0.0) | 2 (0.5) |  |
| NX | 10 (2.0) | 2 (1.7) | 8 (2.2) |  |
| StageM (%) | |  |  | 0.001 |
| M0 | 327 (66.6) | 89 (73.6) | 238 (64.3) |  |
| M1 | 25 (5.1) | 11 (9.1) | 14 (3.8) |  |
| MX | 139 (28.3) | 21 (17.4) | 118 (31.9) |  |
| Stage (%) |  |  |  | <0.001 |
| I | 266 (54.2) | 40 (33.1) | 226 (61.1) |  |
| II | 119 (24.2) | 34 (28.1) | 85 (23.0) |  |
| III | 80 (16.3) | 36 (29.8) | 44 (11.9) |  |
| IV | 26 (5.3) | 11 (9.1) | 15 (4.1) |  |

**Table S2** The cell types identified by marker genes.

| Gene | CellType | Gene | CellType |
| --- | --- | --- | --- |
| EPCAM | Epithelial cells | NKG7 | NK cells |
| KRT19 | Epithelial cells | GNLY | NK cells |
| KRT18 | Epithelial cells | NCAM1 | NK cells |
| CDH1 | Epithelial cells | KLRD1 | NK cells |
| DCN | Fibroblasts | CD79A | B lymphocytes |
| THY1 | Fibroblasts | IGHM | B lymphocytes |
| COL1A1 | Fibroblasts | IGHG3 | B lymphocytes |
| COL1A2 | Fibroblasts | IGHA2 | B lymphocytes |
| PECAM1 | Endothelial cells | LYZ | Myeloid cells |
| CLDN5 | Endothelial cells | MARCO | Myeloid cells |
| FLT1 | Endothelial cells | CD68 | Myeloid cells |
| RAMP2 | Endothelial cells | FCGR3A | Myeloid cells |
| CD3D | T lymphocytes | KIT | MAST cells |
| CD3E | T lymphocytes | MS4A2 | MAST cells |
| CD3G | T lymphocytes | GATA2 | MAST cells |
| TRAC | T lymphocytes |  |  |

**Table S3** GO enrichment analysis in scRNA-seq

| ONTOLOGY | ID | Description | Count | | pvalue |
| --- | --- | --- | --- | --- | --- |
| BP | GO:0010498 | proteasomal protein catabolic process | | 370 | 6.92E-44 |
| BP | GO:0043161 | proteasome-mediated ubiquitin-dependent protein catabolic process | | 308 | 2.76E-36 |
| BP | GO:0016032 | viral process | | 303 | 3.31E-32 |
| BP | GO:0016236 | macroautophagy | | 235 | 1.58E-31 |
| BP | GO:0031331 | Positive regulation of cellular catabolic process | | 319 | 3.60E-31 |
| CC | GO:0005925 | focal adhesion | | 360 | 3.06E-72 |
| CC | GO:0030055 | cell-substrate junction | | 362 | 9.54E-69 |
| CC | GO:0044391 | ribosomal subunit | | 161 | 3.71E-39 |
| CC | GO:0005774 | vacuolar membrane | | 323 | 1.42E-33 |
| CC | GO:0101002 | ficolin-1-rich granule | | 160 | 2.19E-33 |
| MF | GO:0045296 | cadherin binding | | 267 | 4.01E-37 |
| MF | GO:0044389 | ubiquitin-like protein ligase binding | | 239 | 4.65E-26 |
| MF | GO:0031625 | ubiquitin protein ligase binding | | 227 | 6.93E-26 |
| MF | GO:0003735 | structural constituent of ribosome | | 152 | 9.50E-26 |
| MF | GO:0140297 | DNA-binding transcription factor binding | | 329 | 1.85E-25 |

**Table S4** KEGG enrichment analysis in scRNA-seq

| ONTOLOGY | Description | Count | pvalue | |
| --- | --- | --- | --- | --- |
| hsa04141 | Protein processing in endoplasmic reticulum | 146 | | 6.86E-22 |
| hsa05132 | Salmonella infection | 196 | | 2.20E-20 |
| hsa04932 | Non-alcoholic fatty liver disease | 130 | | 3.17E-18 |
| hsa04144 | Endocytosis | 188 | | 1.58E-15 |
| hsa03010 | Ribosome | 127 | | 6.48E-15 |
| hsa04510 | Focal adhesion | 153 | | 7.07E-14 |
| hsa03040 | Spliceosome | 118 | | 7.22E-14 |
| hsa05130 | Pathogenic Escherichia coli infection | 149 | | 3.77E-13 |
| hsa05135 | Yersinia infection | 110 | | 5.00E-13 |
| hsa04210 | Apoptosis | 109 | | 8.01E-13 |
| hsa05171 | Coronavirus disease - COVID-19 | 170 | | 1.02E-12 |
| hsa04142 | Lysosome | 106 | | 1.31E-12 |
| hsa05169 | Epstein-Barr virus infection | 151 | | 1.36E-12 |
| hsa04140 | Autophagy - animal | 111 | | 4.48E-12 |
| hsa05163 | Human cytomegalovirus infection | 164 | | 5.43E-12 |

**Table S5** The intersection of 129 DEGs between TCGA-LUAD and sing-cell RNA-seq

| Gene | logFC | adj.P.Val | Change |
| --- | --- | --- | --- |
| MSR1 | -2.57743 | 1.81702E-61 | DOWN |
| PHLDA2 | 2.235373 | 4.63241E-17 | UP |
| VSIG4 | -2.73029 | 1.02737E-58 | DOWN |
| KLF4 | -2.62059 | 1.92657E-59 | DOWN |
| MS4A7 | -2.35204 | 3.48476E-65 | DOWN |
| MDK | 2.628591 | 2.15971E-21 | UP |
| A2M | -2.00813 | 6.74051E-46 | DOWN |
| FOSB | -3.86461 | 1.20266E-50 | DOWN |
| FABP5 | -2.2256 | 6.32857E-45 | DOWN |
| C15orf48 | 2.391566 | 4.03341E-13 | UP |
| CFD | -2.91585 | 5.15995E-57 | DOWN |
| MARCO | -3.99169 | 5.65465E-70 | DOWN |
| CXCL2 | -2.15363 | 2.87135E-28 | DOWN |
| SLPI | -2.44314 | 9.7753E-26 | DOWN |
| SFTPA1 | -4.298 | 8.17306E-45 | DOWN |
| SFTPA2 | -4.19356 | 1.47878E-43 | DOWN |
| SFTPD | -3.74735 | 5.34494E-51 | DOWN |
| SCGB3A1 | -3.09706 | 2.35973E-17 | DOWN |
| MCEMP1 | -5.10912 | 5.7434E-106 | DOWN |
| SFTPC | -7.8762 | 2.3783E-117 | DOWN |
| COL1A1 | 2.793641 | 1.27287E-15 | UP |
| TPSB2 | -2.47231 | 3.16944E-24 | DOWN |
| RETN | -4.22416 | 3.86723E-61 | DOWN |
| SCGB3A2 | -3.07597 | 6.63155E-18 | DOWN |
| SPP1 | 4.686821 | 4.48239E-21 | UP |
| FABP4 | -6.18696 | 7.7732E-150 | DOWN |
| TPSAB1 | -2.37014 | 2.26749E-29 | DOWN |
| IGKC | 2.258634 | 8.967E-10 | UP |
| SCGB1A1 | -5.94834 | 2.89599E-52 | DOWN |
| IGHG4 | 3.281216 | 1.45699E-12 | UP |
| IGHG1 | 2.747299 | 3.27768E-10 | UP |
| IGHG3 | 2.352727 | 1.2477E-09 | UP |
| ALOX5AP | -2.21214 | 1.58219E-47 | DOWN |
| MRC1 | -2.8644 | 8.86148E-55 | DOWN |
| OLR1 | -2.95435 | 6.8752E-65 | DOWN |
| ALOX5 | -2.01903 | 3.12291E-48 | DOWN |
| GLIPR2 | -2.3438 | 2.14875E-78 | DOWN |
| PHACTR1 | -2.5267 | 4.92174E-70 | DOWN |
| C5AR1 | -2.1215 | 1.39937E-57 | DOWN |
| DOK2 | -2.15465 | 5.39828E-44 | DOWN |
| RBP4 | -3.40296 | 7.47563E-47 | DOWN |
| HBEGF | -2.69864 | 2.17363E-77 | DOWN |
| CD52 | -2.58152 | 2.8162E-60 | DOWN |
| EMP2 | -2.86281 | 1.696E-121 | DOWN |
| C4BPA | -2.23079 | 2.4354E-15 | DOWN |
| CDKN2A | 2.048906 | 1.72788E-07 | UP |
| CRABP2 | 4.499874 | 8.63825E-17 | UP |
| SFTA1P | -3.20616 | 2.2946E-39 | DOWN |
| ABCC3 | 2.197018 | 3.19907E-17 | UP |
| C11orf96 | -2.00789 | 5.00969E-33 | DOWN |
| SPINK1 | 4.6055 | 1.03787E-09 | UP |
| AQP1 | -2.69454 | 3.79645E-41 | DOWN |
| PLPP2 | 2.630466 | 5.43642E-34 | UP |
| SLC39A8 | -2.7821 | 3.2848E-99 | DOWN |
| NME1 | 2.068511 | 8.90904E-32 | UP |
| LRRK2 | -3.04957 | 1.68338E-43 | DOWN |
| CEACAM5 | 3.621131 | 9.14189E-08 | UP |
| TPPP3 | -3.07832 | 1.56885E-51 | DOWN |
| RGCC | -2.96204 | 1.9787E-124 | DOWN |
| FGFBP2 | -3.96752 | 3.30306E-77 | DOWN |
| IGHG2 | 2.512744 | 7.56751E-11 | UP |
| IGHGP | 2.560037 | 2.45632E-09 | UP |
| DERL3 | 2.019533 | 3.76478E-15 | UP |
| IGLV6-57 | 2.092183 | 1.14966E-07 | UP |
| VPREB3 | 2.112666 | 5.94996E-14 | UP |
| CPA3 | -2.33767 | 5.68228E-28 | DOWN |
| MS4A2 | -2.52912 | 8.50213E-33 | DOWN |
| CTSG | -2.38194 | 2.99417E-20 | DOWN |
| RGS13 | -2.0162 | 8.16163E-27 | DOWN |
| CLDN5 | -3.09133 | 1.96105E-68 | DOWN |
| SPARCL1 | -2.11417 | 1.05478E-58 | DOWN |
| GNG11 | -2.07938 | 4.99987E-45 | DOWN |
| RAMP2 | -3.05053 | 2.2467E-103 | DOWN |
| ACKR1 | -2.96011 | 6.07869E-37 | DOWN |
| VWF | -2.54043 | 4.43325E-71 | DOWN |
| CAV1 | -4.03179 | 2.8941E-140 | DOWN |
| CLEC14A | -2.41458 | 1.18219E-90 | DOWN |
| ECSCR | -2.35055 | 3.27596E-68 | DOWN |
| PECAM1 | -2.41053 | 3.5642E-122 | DOWN |
| EPAS1 | -2.63739 | 5.764E-120 | DOWN |
| RAMP3 | -3.26226 | 5.9031E-99 | DOWN |
| FCN3 | -4.8548 | 3.8646E-106 | DOWN |
| CD93 | -2.30333 | 2.00086E-79 | DOWN |
| ESAM | -2.23229 | 5.20385E-65 | DOWN |
| LDB2 | -2.69933 | 1.8703E-114 | DOWN |
| CALCRL | -2.86929 | 2.9282E-101 | DOWN |
| EMCN | -2.99531 | 3.25147E-92 | DOWN |
| PCAT19 | -2.53358 | 2.77076E-75 | DOWN |
| DNASE1L3 | -3.88413 | 4.1437E-69 | DOWN |
| PTPRB | -2.74536 | 1.74756E-88 | DOWN |
| IL33 | -2.68706 | 9.69397E-53 | DOWN |
| CDH5 | -2.66248 | 5.6361E-111 | DOWN |
| TSPAN7 | -2.7932 | 2.847E-45 | DOWN |
| PALMD | -2.26769 | 2.55503E-67 | DOWN |
| CAV2 | -2.7766 | 3.37964E-79 | DOWN |
| JAM2 | -2.67388 | 3.3516E-112 | DOWN |
| CYYR1 | -2.32376 | 6.2588E-95 | DOWN |
| ROBO4 | -2.98841 | 1.0827E-111 | DOWN |
| MMRN1 | -2.64865 | 1.05577E-47 | DOWN |
| TIE1 | -2.14633 | 7.4029E-75 | DOWN |
| ACVRL1 | -2.62167 | 1.5455E-121 | DOWN |
| NRN1 | -2.23762 | 2.94581E-45 | DOWN |
| HEG1 | -2.26485 | 2.95319E-77 | DOWN |
| TIMP3 | -2.14088 | 8.76343E-52 | DOWN |
| GPX3 | -2.69037 | 2.58386E-77 | DOWN |
| COL3A1 | 2.417545 | 5.77692E-17 | UP |
| MFAP4 | -3.17333 | 8.74546E-71 | DOWN |
| C7 | -2.69566 | 5.79324E-30 | DOWN |
| CTHRC1 | 3.725019 | 8.06016E-33 | UP |
| MMP11 | 4.832919 | 8.69651E-23 | UP |
| ADH1B | -4.78375 | 1.15029E-68 | DOWN |
| PLAC9 | -2.8328 | 3.97983E-95 | DOWN |
| FHL1 | -3.93421 | 1.0867E-126 | DOWN |
| THBS2 | 2.540273 | 9.07484E-17 | UP |
| TCF21 | -3.83398 | 6.54534E-88 | DOWN |
| PPP1R14A | -2.25152 | 2.01373E-40 | DOWN |
| GPC3 | -3.0023 | 1.52601E-56 | DOWN |
| FXYD1 | -3.03356 | 3.32236E-38 | DOWN |
| INMT | -4.15576 | 1.8208E-103 | DOWN |
| PDLIM2 | -2.12461 | 1.36466E-85 | DOWN |
| FBLN5 | -2.16507 | 1.01714E-54 | DOWN |
| FMO2 | -3.63102 | 6.99473E-98 | DOWN |
| COL10A1 | 4.627847 | 1.31273E-29 | UP |
| LTBP4 | -2.40373 | 8.53157E-67 | DOWN |
| FXYD6 | -2.03505 | 1.70158E-57 | DOWN |
| CYBRD1 | -2.1065 | 5.22739E-52 | DOWN |
| CRYAB | -2.35051 | 8.07073E-58 | DOWN |
| COMP | 2.645227 | 4.01552E-10 | UP |
| CXCL14 | 2.42721 | 6.11631E-07 | UP |

**Table S6** GO enrichment analysis of the intersecting genes between bulk and single-cell transcription

| ONTOLOGY | ID | Description | | Count | | pvalue |
| --- | --- | --- | --- | --- | --- | --- |
| BP | GO:0006959 | | humoral immune response | | 19 | 1.75E-13 |
| BP | GO:0006956 | | complement activation | | 13 | 2.58E-12 |
| BP | GO:0001894 | | protein lipoylation | | 15 | 2.38E-10 |
| BP | GO:0030198 | | cellular copper ion homeosta-sis | | 15 | 1.27E-09 |
| BP | GO:0043062 | | copper ion transmembrane transport | | 15 | 1.33E-09 |
| CC | GO:0062023 | | collagen-containing extracellular matrix | | 23 | 3.65E-15 |
| CC | GO:0005581 | | mitochondrial pyruvate dehydrogenase complex | | 10 | 1.82E-10 |
| CC | GO:1903143 | | adrenomedullin receptor complex | | 3 | 2.53E-07 |
| CC | GO:1903439 | | calcitonin family receptor complex | | 3 | 2.51E-06 |
| CC | GO:0072562 | | blood microparticle | | 8 | 4.65E-06 |
| MF | GO:0005201 | | extracellular matrix structural constituent | | 11 | 1.51E-08 |
| MF | GO:0001605 | | adrenomedullin receptor activity | | 3 | 2.63E-07 |
| MF | GO:0002020 | | protease binding | | 8 | 2.84E-06 |
| MF | GO:0097642 | | pyruvate dehydrogenase activity | | 3 | 5.19E-06 |
| MF | GO:0005178 | | integrin binding | | 8 | 7.86E-06 |

**Table S7** Univariate and multivariate cox survival analyses

| ID | Univariate analysis | | | Multivariate analysis | | |
| --- | --- | --- | --- | --- | --- | --- |
|  | HR | 95%CI of HR | pvalue | HR | 95%CI of HR | pvalue |
| KLF4 | 1.44 | 1.17-1.78 | 0.0006 | 1.57 | 1.27-1.95 | 0.0000 |
| SCGB3A1 | 0.90 | 0.84-0.97 | 0.0099 | 0.90 | 0.83-0.97 | 0.0079 |
| COL1A1 | 1.26 | 1.07-1.47 | 0.0044 | 1.17 | 1.00-1.38 | 0.0492 |
| SPP1 | 1.19 | 1.05-1.34 | 0.0064 |  |  |  |
| C4BPA | 0.86 | 0.78-0.96 | 0.0057 |  |  |  |
| TSPAN7 | 0.80 | 0.67-0.96 | 0.0155 | 0.83 | 0.68-1.00 | 0.0521 |
| CAV2 | 1.32 | 1.07-1.62 | 0.0103 |  |  |  |
| CTHRC1 | 1.29 | 1.07-1.55 | 0.0073 |  |  |  |
