# Supplementary figures and images for: Identification and validation of a novel cuproptosis-related stemness signature to predict prognosis and immune landscape in lung adenocarcinoma by integrating single-cell and bulk RNA-sequencing
Source: Front Immunol. 2023 May 23;14:1174762. doi: 10.3389/fimmu.2023.1174762 (PMC10242006; doi:10.3389/fimmu.2023.1174762)

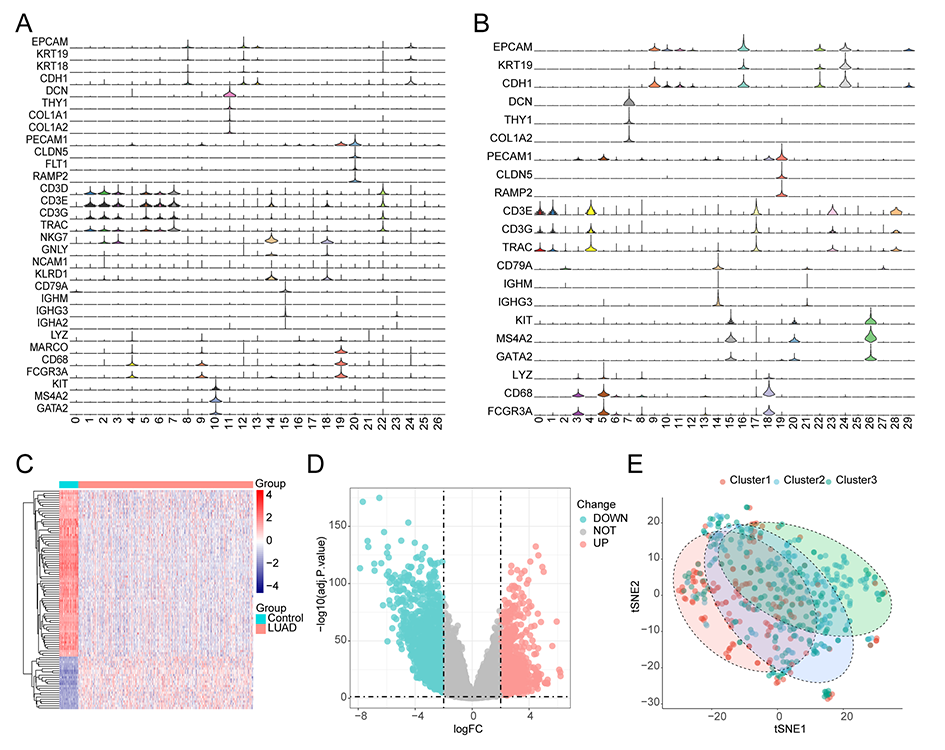

Supplement: Supplementary file 2 [file Image_1.tif]

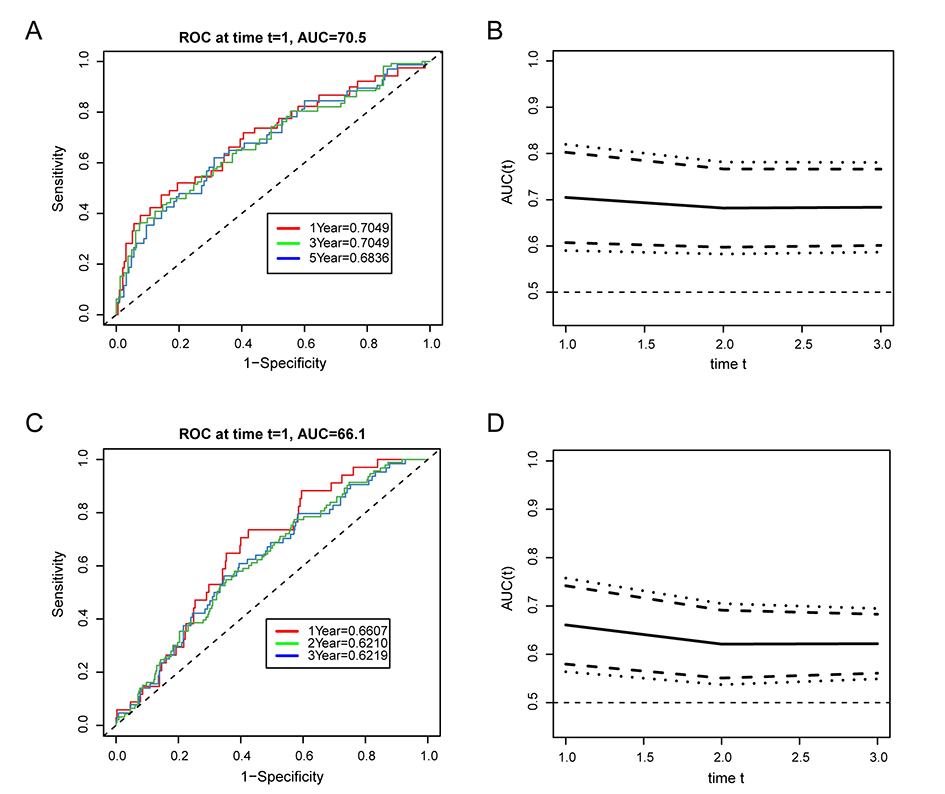

Supplement: Supplementary file 3 [file Image_2.tif]

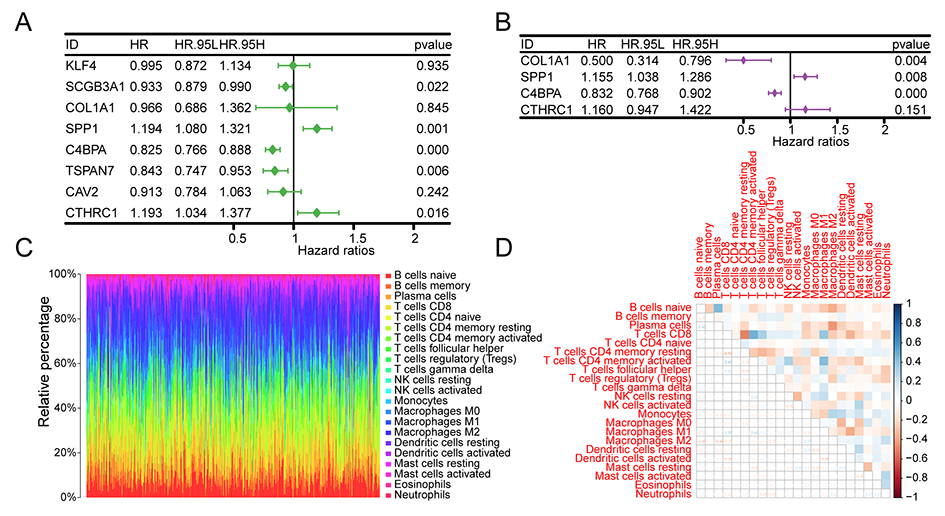

Supplement: Supplementary file 4 [file Image_3.tif]

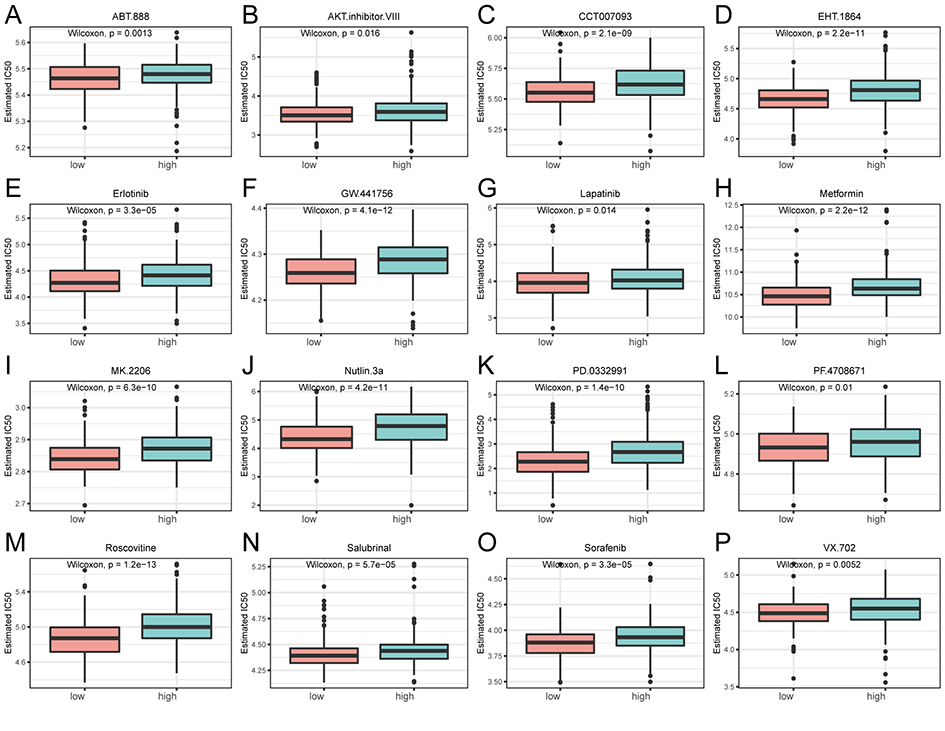

Supplement: Supplementary file 5 [file Image_4.tif]
